# The brain antigen-specific B cell response correlates with glatiramer acetate responsiveness in relapsing-remitting multiple sclerosis patients

**DOI:** 10.1038/srep14265

**Published:** 2015-09-21

**Authors:** Damiano M. Rovituso, Cathrina E. Duffy, Michael Schroeter, Claudia C. Kaiser, Christoph Kleinschnitz, Antonios Bayas, Rebecca Elsner, Stefanie Kuerten

**Affiliations:** 1 Department of Anatomy and Cell Biology, University of Würzburg, Würzburg, Germany; 2 Department of Anatomy I, University of Cologne, Cologne, Germany; 3 Department of Neurology, University Hospitals of Cologne, Cologne, Germany; 4 Department of Neurology, University Hospitals of Würzburg, Würzburg, Germany; 5 Department of Neurology, Klinikum Augsburg, Augsburg, Germany; 6 NeuroCure Clinical Research Center (NCRC), Charité-Universitätsmedizin Berlin, Berlin, Germany

## Abstract

B cells have only recently begun to attract attention in the immunopathology of multiple sclerosis (MS). Suitable markers for the prediction of treatment success with immunomodulatory drugs are still missing. Here we evaluated the B cell response to brain antigens in n = 34 relapsing-remitting MS (RRMS) patients treated with glatiramer acetate (GA) using the enzyme-linked immunospot technique (ELISPOT). Our data demonstrate that patients can be subdivided into responders that show brain-specific B cell reactivity in the blood and patients without this reactivity. Only in patients that classified as B cell responders, there was a significant positive correlation between treatment duration and the time since last relapse in our study. This correlation was GA-specific because it was absent in a control group that consisted of interferon-ß (IFN-β)-treated RRMS patients (*n* = 23). These data suggest that GA has an effect on brain-reactive B cells in a subset of patients and that only this subset benefits from treatment. The detection of brain-reactive B cells is likely to be a suitable tool to identify drug responders.

Multiple sclerosis (MS) is a chronic, inflammatory demyelinating disease of the central nervous system (CNS) characterized by an initial inflammatory phase, followed by selective demyelination and neurodegeneration^1^. Recently published studies support the hypothesis that inflammatory cortical demyelinating processes are closely related to the onset of the disease and that disease progression could be explained by myelin-laden macrophages that leave the CNS compartment to enter the cervical lymph nodes where they perpetuate inflammation^2^. Studies of early active MS lesions reported interindividual heterogeneity, but intraindividual homogeneity in the patterns of demyelinating plaque pathology^3^,^4^, where the most frequently observed pattern was selectively associated with immunoglobulin and complement deposition^3^. Contrasting this notion, it has been proposed that all MS lesions begin with oligodendrocyte apoptosis, followed by antibody deposition and complement activation^5^. Breij *et al.* reported that antibody- and complement-mediated myelin phagocytosis was the dominant mechanism in all chronic MS lesions^6^. Moreover, it has been suggested that heterogeneity of disease was found in early stages of lesion formation, but was absent in established MS^6^. The role of B cells in the pathology of MS has largely been underestimated in the past. Recently, Disanto *et al.* delineated that the current knowledge on B cell involvement in MS nearly fulfilled all nine Hill’s criteria for causation^7^. Indeed, throughout the disease course B cells and antibodies play a pivotal role. On the one hand, the presence of anti-myelin antibodies predicted the second clinical episode within three years after the first demyelinating event^8^. On the other hand, meningeal germinal center-like structures were associated with a more severe disease course, an earlier age at MS onset and a more rapid conversion to progressive disease and death^9^,^10^.

Glatiramer acetate (GA) is an approved first-line drug for the immunomodulatory treatment of MS and composed of alanine, glutamic acid, lysine and tyrosine. It is thought to act as an altered peptide ligand to inhibit myelin basic protein-specific T cells^11^. A pivotal mechanism of action is the induction of anti-inflammatory cytokines, produced by T helper (T_H_) cells and B cells, leading to “bystander suppression” at the site of focal inflammation^12^. Furthermore, GA-specific antibodies have been identified in GA-treated MS patients^13^,^14^. Remarkably, the level of GA-specific antibodies of the T_H_2-associated IgG4 isotype was inversely correlated with the number of relapses, but only in long-term treatment^15^. These results suggest that GA treatment responsiveness could be monitored by an antibody assay. GA therapy was shown to remodel the composition of the B cell compartment and to influence cytokine secretion and immunoglobulin production^16^. These aforementioned effects on B cells could help to characterize a more B cell-driven MS phenotype and elucidate a novel mechanism of action. Additionally, biomarkers that predict the therapeutic benefit of a MS drug need to be developed in order to accurately differentiate between treatment responders and non-responders. However, to date there is no such biomarker.

Interferon-β (IFN-β) is also a first-line disease modifying drug for the treatment of RRMS^17^. Its mechanisms of action are not fully understood yet, but it has been shown that IFN-β alters cytokine production in T cells^18^, enhances apoptosis of T_H_17 cells *in vitro* and reduces the percentage of T_H_17 cells in relapsing-remitting MS (RRMS) patients^19^. B cells are also targeted by IFN-β in their cytokine production in a way that inhibits T_H_17 cell differentiation^20^. Furthermore, it was shown that B cell survival and differentiation are affected through IFN-β-mediated induction of the B cell activating factor of the TNF family (BAFF)^21^. Recent findings indicate that IFN-β increases the number of CD19^+^CD24^++^CD38^++^ transitional B cells, which in turn suppress the differentiation of CD4^+^ T cells^22^,^23^.

We have previously introduced an enzyme-linked immunospot technique (ELISPOT) assay for the *ex vivo* detection of brain-specific B cells^24^,^25^. Brain-reactive B cells were only detected in patients with clinically isolated syndrome or MS, but were absent in healthy subjects or in patients with other neurological or autoimmune diseases^24^,^25^. We have now used this bioassay to investigate whether GA treatment has an influence on the presence of autoreactive B cells in the blood of RRMS patients, and *vice versa*. First, we addressed the question if the presence of a brain-specific B cell response in the ELISPOT assay was reflective of GA treatment responsiveness. Second, to evaluate whether these findings were specific for GA we also included IFN-β-treated patients in our study. Finally, we compared the brain-specific B cell response in patients with a different disability status.

## Results

### Characteristics of the MS patient population

All patients in this study were diagnosed with RRMS. We classified patients with spot counts >4.5 as ELISPOT responders and patients with spot counts ≤4.5 as ELISPOT non-responders as described before^24^. In the GA-treated group, the mean (±standard deviation) age was 38.50 (±11.75) years and the mean treatment duration was 20.35 (±14.73) months (Table 1). We characterized *n* = 22 GA-treated RRMS patients as ELISPOT responders and *n* = 12 as ELISPOT non-responders. We separately examined GA treatment duration, the brain-reactive B cell response and the time since last relapse with respect to the disability score of the patients. In addition, we tested *n* = 23 RRMS patients that were treated with IFN-β (Table 2). The mean (±standard deviation) age was 41.09 (±9.59) years and the mean treatment duration was 29.35 (±28.58) months. *N* = 10 IFN-ß-treated RRMS were ELISPOT responders, while *n* = 13 patients were non-responders. Following Leray *et al.* we used an expanded disability status scale (EDSS) > 3 as a threshold of irreversible disability and subsequently we classified a disability score from EDSS 0 to 2.5 as “mild” and from 3 to 6 as “severe” disability (Table 3)^26^.

### The presence of brain antigen-specific B cells in the blood of RRMS patients correlates with GA responsiveness

**Figure 1:**
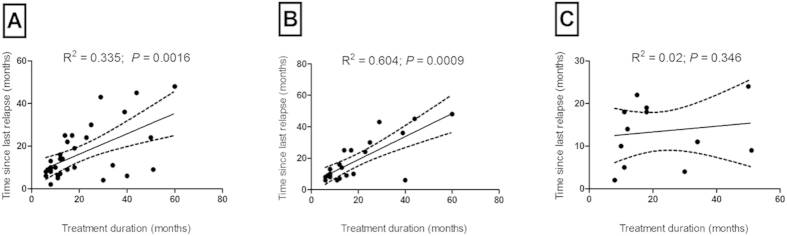
Correlation between glatiramer acetate (GA) treatment and the time since last relapse. (**A**) Correlation between the treatment duration (months) and the time since last relapse (months) in all patients with relapsing-remitting multiple sclerosis (RRMS) (*n* = 34, *P* < 0.002). (**B**) Correlation between the treatment duration (months) and the time since last relapse (months) in RRMS patients with brain-specific B cells in the blood (ELISPOT responders, *n* = 22, *P* < 0.001). (**C**) Absence of correlation between treatment duration (months) and the time since last relapse (months) in RRMS patients without brain-specific B cells in the blood (ELISPOT non-responders, *n* = 12, *P* = 0.35).

**Figure 2:**
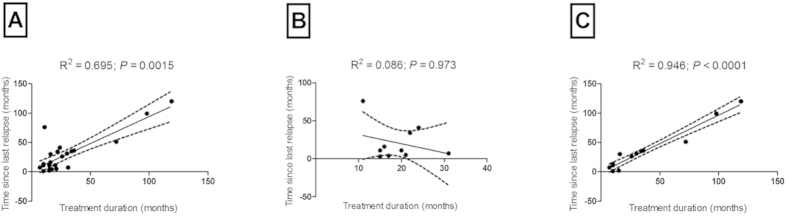
Correlation between interferon-β (IFN-β) treatment and the time since last relapse. (**A**) Correlation between the treatment duration (months) and the time since last relapse (months) in all patients with relapsing-remitting multiple sclerosis (RRMS) (*n* = 23, *P* = 0.0015). (**B**) Absence of correlation between the treatment duration (months) and the time since last relapse (months) in RRMS patients with brain-specific B cells in the blood (ELISPOT responders, *n* = 10, *P* = 0.973). (**C**) Correlation between treatment duration (months) and the time since last relapse (months) in RRMS patients without brain-specific B cells in the blood (ELISPOT non-responders, *n* = 13, *P* = 0.0001).

In two randomized, placebo-controlled studies GA reduced the annualized relapse rate and progression of disability, as measured by the EDSS in patients with RRMS^27^,^28^. As expected, we were able to assess a strong positive correlation between the treatment duration and the time since last relapse in GA-treated RRMS patients in our study (*r*_s_ = 0.53, *P* < 0.002; Fig. 1A). Considering the B cell response to brain antigen as a categorical variable, we looked for a correlation between the aforementioned clinical parameters and found that the ELISPOT responders (*n* = 22) showed a strong positive correlation between the treatment duration and the time since last relapse (*r*_s_ = 0.66, *P* < 0.001; Fig. 1B). On the contrary, there was no correlation between treatment duration and the time since last relapse in the ELISPOT non-responder group (*n* = 12, *r*_s_ = 0.28, *P* = 0.35; Fig. 1C). Moreover, we evaluated the association between treatment duration and the presence of brain-reactive B cells in the ELISPOT responder group (*n* = 22). We observed no correlation between these two parameters (*r*_s_ = 0.007, *P* = 0.97). The same applied to the association between the time since last relapse and the brain-reactive B cell response in the ELISPOT responder group (*r*_s_ = −0.159, *P* = 0.48). When comparing the time since last relapse, EDSS, treatment duration and age no differences between ELISPOT responders and non-responders were evident (TSLR *P* = 0.379; EDSS *P* = 0.847; treatment duration *P* = 0.212 and age *P* = 0.376). In several studies it was shown that GA treatment induced GA-specific antibodies. Brenner *et al.* reported that GA-treated MS patients with high GA-reactive antibody titers were more likely to be relapse-free than patients with lower GA-reactive antibody titers^13^. Controversial findings reported no association between GA-specific antibody titers and clinical outcomes^29^. In addition, new data suggest that the effects of GA on cytokine production by human B cells are donor-specific and that the interaction with the B cell receptor is required for GA efficacy at least in a murine model^30^. Based on these previous results we set out to investigate if our findings were GA-specific and therefore we also tested IFN-β-treated RRMS patients. As expected, our data demonstrate a strong positive correlation between treatment duration and the time since last relapse in RRMS patients (*n* = 23) that were treated with IFN-β (*r*_s_ = 0.62, *P* < 0.002; Fig. 2A). In contrast to the GA-treated cohort, the ELISPOT non-responders displayed a very strong correlation (*n* = 13, *r*_s_ = 0.93, *P* = 0.0001; Fig. 2B) between the aforementioned parameters, while the ELISPOT responders did not show any relationship between treatment duration and the time since last relapse (*n* = 10, *r*_s_ = 0.01, *P* = 0.97; Fig. 2B). When comparing the time since last relapse, treatment duration and age no differences between ELISPOT responders and non-responders were evident in the IFN-β-treated cohort (TSLR *P* = 0.352; treatment duration *P* = 0.619 and age *P* = 0.437).

### GA treatment affects the frequency of brain-specific B cells in the periphery only during an early stage of MS

**Figure 3:**
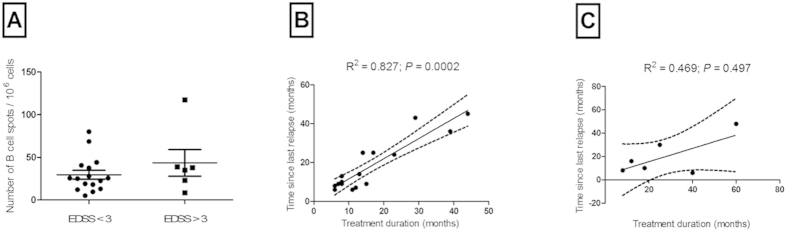
Association between the disability status and the brain-reactive B cell response in relapsing-remitting multiple sclerosis (RRMS) patients. (**A**) Comparison of the spot counts between GA-treated RRMS patients with a disability status <3 (*n* = 16) and >3 (*n* = 6). The graph displays means and standard deviations. (**B**) Correlation between the treatment duration and the time since last relapse in RRMS patients with a brain-reactive B cell response and a mild disability status (EDSS < 3; *P* < 0.001). (**C**) Correlation between treatment duration and the time since last relapse in RRMS patients with a brain-reactive B cells response and a severe disability status (EDSS > 3; *P* = 0.49).

It has been reported that relapses during the first two years of disease were predictive of the late disease outcome, whereas late relapses did not seem to affect the prognosis^31^. Accordingly, the exacerbation rate at an early phase of the disease was demonstrated to enhance the accumulation of disability^32^. The assumption that MS follows a two-stage process was further supported by Leray *et al.* (2010). Leray *et al.* reported that disability progression during the late phase was independent of that during the early phase. However, relapses during the first two years in the early phase were independent predictive factors of disability progression^26^. When we analyzed the number of brain-reactive B cells in GA-treated RRMS patients with a mild disease course (EDSS ≤ 3; *n* = 16) and more severe disease (EDSS ≥ 3; *n* = 6), we did not find any difference (*P* = 0.44; Fig. 3A). However, when we evaluated the ELISPOT responder group in the light of their disability status we found a strong association between the treatment duration and the time since last relapse in the cohort with a mild course (*n* = 16, *r*_s_ = 0.79, *P* < 0.001; Fig. 3B), while we could not observe a correlation between the aforementioned parameters in patients who suffered from a more severe disease course (*n* = 6, *r*_s_ = 0.37, *P* = 0.49; Fig. 3C).

## Discussion

As previously reported, we were able to identify MS patients with a brain-reactive B cell response in the blood^24^,^25^. We hypothesized that the identification of brain-specific B cells in RRMS patients is as crucial at an early stage of the disease as the identification of treatment responders at the onset of GA therapy. GA treatment does not only induce antibodies against GA, but it also increases anti-histone antibodies^33^. There have been several efforts to identify drug responders and non-responders by using a standard proliferation assay combined with ELISPOT^14^. The immunological response profile, including the ELISPOT response to IFN-γ and IL-4, correlated with the clinical response to GA treatment^34^. More importantly, an increased IL-4/IFN-γ ratio was associated with a milder clinical response in stimulated peripheral blood mononuclear cells (PBMC) of GA-treated patients^35^.

In this report, the major new observation is that the presence of brain-specific B cells is a categorical variable to identify GA responders. First, we found that the therapeutic benefit from GA treatment was greater in RRMS patients who displayed brain antigen-reactive B cells in the blood compared to patients without. Second, a brain-antigen specific response in our bioassay was associated with drug responsiveness only in GA-treated patients, while this association was absent in the IFN-β-treated cohort. Interestingly, in the IFN-β-treated group the patients characterized by the absence of brain-reactive B cells showed a benefit from the treatment, suggesting that IFN-β effectiveness might be compromised in patients with brain-reactive B cells. We assume that the B cells that produce brain-reactive antibodies are distinct from the B cell subpopulations that are susceptible to IFN-β treatment. One mechanism of action of IFN-β on B cells is the downregulation of CD40^20^ resulting in impaired cognate B-T cell interaction. It is conceivable that the brain-reactive B cells as detected by our assay are not affected by this mechanism, while they are modulated by GA treatment.

Overall, our data also strongly support the concept of heterogeneity in the immunopathology of MS as described before^3^. Histological analysis revealed that lesions within one patient were similarly composed, while four general subtypes were identified among different patients^3^. Patients with the most frequent lesion pattern that was characterized by antibody deposition and complement activation positively responded to therapeutic plasma exchange in acute relapses, whereas patients without antibody deposition failed to do so^36^. These findings imply that the heterogeneity found histologically reflected a similar heterogeneity in the pathophysiology of the disease. Conversely, recent studies suggested that heterogeneity of the disease was mostly found in the early stages of lesion formation, while being absent in established MS^6^. Third, we focused on the disability status and compared patients with a mild and severe disability status. Our data may provide evidence that once a clinical threshold of irreversible disability has been reached GA does not have the same benefit as for patients at an early stage of disease. In this context it is of note that in two former trials in RRMS GA was more effective in patients with an EDSS score between 0 and 2^27^,^28^. GA efficacy in patients with more severe disease might indeed be affected by the natural history and progression of the disease. We do not question this possibility. However, it was our aim to show that treatment with GA at an early disease stage is very important for its clinical efficacy, while the EDSS is not as suitable to categorize drug responsiveness as our B cell ELISPOT. Yet, we are aware of the fact that the assumption of a correlation between GA responsiveness, the brain-reactive B cell response and disease activity is not necessarily a causal one. At this point, our data are limited in that they do not show a direct effect of GA on B cells. Nevertheless, the B cell response should still be a discriminating factor for treatment responsiveness as demonstrated in this study and not solely an indicator of disease activity, while the EDSS will be useful to investigate the two-stage history of RRMS. We suggest that combining the information on both the disease stage and responsiveness to brain antigens will be crucial for an accurate and individual MS therapy. Our data support the notion of a two-stage disability process as suggested by Leray *et al.*^26^. We hypothesize that B cells could play a role in a more progressive disease as reported before^9^ and one could hypothesize that MS patients who display brain antigen-reactive B cells in the blood are more likely to show disease progression. We also suggest that B cells from patients with a more severe disability status are less likely to be remodelled in contrast to B cells from patients with a milder disability status. In addition, we postulate that RRMS patients displaying brain-reactive B cells are likely to benefit from GA treatment through a novel recently described mechanism of action that targets anti-inflammatory B cell properties^16^. In summary, we have introduced a simple bioassay to identify GA-treated MS patients that show B cell responsiveness to brain antigens in the blood. These results could have far-reaching implications for future treatment strategies of MS.

## Methods

### Study participants

PBMC were obtained from MS patients that were treated with GA (*n* = 34) or IFN-β (*n* = 23) for at least six months. MS diagnosis was established according to the 2005 McDonald criteria. All patients additionally fulfilled the 2010 revised criteria^37^. Patients with a history of other autoimmune diseases and severe accompanying systemic or psychiatric disorders were excluded from the study. Likewise, patients that had undergone plasmapheresis, B cell depletion therapy, intravenous immunoglobulin or immunosuppressive treatment 12 months prior to the inclusion into the study were also excluded. Characteristics of the cohorts are listed in Tables 1, 2 and 3. All experimental protocols were approved by the ethical review committees of the University Hospitals of Cologne and Würzburg, the Charité-Universitätsmedizin Berlin and the Bavarian Chamber of Physicians for the Klinikum Augsburg (approval numbers 10–221, 65/10, 149/11 and mb BO 14043). Written informed consent was obtained from each patient. Disability was graded using the EDSS^38^. The methods were carried out in accordance with the approved guidelines.

### Isolation of PBMC and polyclonal stimulation

PBMC were separated from heparinized blood by density gradient centrifugation and cultured at a concentration of 3 × 10^6^ cells/ml in complete RPMI-1640 supplemented with IL-2 at 15 ng/ml (Peprotech), R-848 at 2.5 μg/ml (Enzo Life Sciences, Inc.) and 1 μmol/L β-mercaptoethanol (Sigma) for 96 h at 37 °C and 7% CO_2_, according to the protocol described by Pinna *et al.*^39^.

### ELISPOT assays

Ninety-six-well PVDF ELISPOT plates (MultiScreen HTS Millipore) were coated overnight with whole human brain lysate (30 μg/ml; Novus Biologicals, Littleton, CO). Anti-human Igκ (SouthernBiothech Biozol) was used as a positive control at a concentration of 10 μg/ml and 10% FBS was used as a medium control. Plates were blocked with sterile 10% FBS for 2 h at room temperature. Each sample was plated in duplicates with 1 × 10^6^ cells/well and incubated at 37 °C and 7% CO_2_ for 26 h. After culture, the plates were incubated with biotinylated anti-human IgG (Hybridoma Reagent Laboratory) at 0.2 μg/ml in 1% BSA. Subsequently, all plates were developed with AP-KIT III substrate (Vector Blue; Vector Laboratories). Spots were counted on an Immunospot^®^ Series 6 Analyzer (Cellular Technology Limited). Brain-reactive antibodies were mainly of the IgG1 and IgG3 isotype (data not shown).

### Statistical analysis

Results were compared between two groups of GA-treated patients using the Mann-Whitney test. A value of *P* < 0.05 was considered as statistically significant. To investigate the relationship between GA treatment duration, ELISPOT response and the time since last relapse, Spearman correlation analyses between two variables were applied and a correlation coefficient (*r*_s_) of *P* < 0.05 (two-sided tests) was considered as statistically significant.

## Additional Information

**How to cite this article**: Rovituso, D. M. *et al.* The brain antigen-specific B cell response correlates with glatiramer acetate responsiveness in relapsing-remitting multiple sclerosis patients. *Sci. Rep.* **5**, 14265; doi: 10.1038/srep14265 (2015).

## Figures and Tables

**Table 1:** Characteristics of glatiramer acetate (GA)-treated relapsing-remitting multiple sclerosis (RRMS) patients.

	**RRMS patients**	**RRMS patients “Responders”**	**RRMS patients “Non-responders”**
Number of patients	34	22	12
Female: male sex ratio	1.84	1.5	3.3
Female	22	12	9
Male	12	8	3
Mean time since last relapse (months) ± SD	16.55 ± 12.30 (*n* = 33)	18.50 ± 13.83	12.64 ± 7.54 (*n* = 11)
Mean EDSS score ± SD	2.23 ± 1.56 (*n* = 33)	2.30 ± 1.74	2.09 ± 1.20 (*n* = 11)
Mean GA treatment duration (months) ± SD	20.35 ± 14.73	19.23 ± 14.65	22.42 ± 15.29

Abbreviations: GA glatiramer acetate; MS multiple sclerosis; RRMS relapsing-remitting multiple sclerosis; SD standard deviation; EDSS expanded disability status scale; Responders/Non-responders: presence/absence of brain-specific antibody production as measured by ELISPOT.

**Table 2:** Characteristics of interferon-β (IFN-β) treated relapsing-remitting multiple sclerosis (RRMS) patients.

	**RRMS patients**	**RRMS patients “Responders”**	**RRMS patients “Non-responders”**
Number of patients	23	10	13
Female: male sex ratio	1.56	1	5.5
Female	14	5	11
Male	9	5	2
Mean time since last relapse (months) ± SD	29.13 ± 31.55	20.80 ± 23.29	35.54 ± 36.27
Mean EDSS score ± SD	1.73 ± 1.25 (*n* = 11)	2 (*n* = 1)	1.70 ± 1.32 (*n* = 10)
Mean IFN-β treatment duration (months) ± SD	29.35 ± 28.58	19.20 ± 5.69	37.15 ± 36.36

Abbreviations: IFN-β interferon-β; MS multiple sclerosis; RRMS relapsing-remitting multiple sclerosis; SD standard deviation; EDSS expanded disability status scale; Responders/Non-responders: presence/absence of brain-specific antibody production as measured by ELISPOT.

**Table 3:** Characteristics of glatiramer acetate (GA)-treated relapsing-remitting multiple sclerosis (RRMS) patients with mild and severe disability score.

	**RRMS patients EDSS < 3**	**RRMS patients EDSS > 3**
Number of patients	16	6
Female: male sex ratio	1.7	1
Female	10	3
Male	6	3
Mean age (years) ± SD	35.13 ± 12.97	44 ± 8.53
Mean time since last relapse (months) ± SD	18.06 ± 13.34	19.67 ± 16.37
Mean EDSS score ± SD	1.47 ± 0.86	4.5 ± 1.55
Mean GA treatment duration (months) ± SD	16.25 ± 11.72	27.17 ± 19.64

Abbreviations: GA glatiramer acetate; MS multiple sclerosis; RRMS relapsing-remitting multiple sclerosis; SD standard deviation; EDSS expanded disability status scale.
